# Non-Melanoma-Associated Dyschromia of the Proximal Nail Fold

**DOI:** 10.7759/cureus.922

**Published:** 2016-12-09

**Authors:** Nicole C DeMartinis, Philip R Cohen

**Affiliations:** 1 School of Medicine, University of California, San Diego; 2 Department of Dermatology, University of California, San Diego

**Keywords:** black, brown, chloronychia, discoloration, dyschromia, hematoma, hutchinson’s sign, nail fold, pseudo-hutchinson’s sign, subungual melanoma

## Abstract

Subungual melanoma with pigmentation beneath the nail that extends to involve the proximal nail fold is referred to as Hutchinson’s sign. Black or brown subungual discoloration involving the proximal nail fold secondary to other etiologies has been referred to as pseudo-Hutchinson’s sign. Three patients with nail discoloration and concurrent dyschromia of the proximal nail fold are described: a female with a chronic subungual hematoma and pseudo-Hutchinson’s sign, a male with culture-confirmed *Pseudomonas aeruginosa* (*P. aeruginosa) *of the nail with green discoloration involving the proximal nail fold, and a male with an acute subungual hematoma with red-purple subungual discoloration affecting the proximal nail fold. PubMed was searched for the following: black, brown, chloronychia, discoloration, dyschromia, green, hematoma, Hutchinson’s sign, nail, nail fold, proximal, pseudo-Hutchinson’s sign, red, subungual melanoma, syndrome. The papers were reviewed and appropriate references evaluated. In conclusion, melanoma-associated black proximal nail fold pigmentation is referred to as Hutchinson’s sign, and non-melanoma-associated black pigmentation has been designated as pseudo-Hutchinson’s sign. Subungual nail plate discoloration extending to involve the proximal nail fold may be black, green, or red-purple in patients with melanocytic and non-melanocytic lesions, bacterial infection (*Pseudomonas*), and acute subungual hematoma, respectively. Instead of creating a new terminology, we suggest that non-black subungual discoloration (green or red-purple) extending to involve the proximal nail fold be referred to as pseudo pseudo-Hutchinson’s sign.

## Introduction

Subungual pigmentation affects the nail and may extend to also involve the nail fold. Hutchinson’s sign describes the presence of black or brown subungual pigmentation including the proximal nail fold [1]. Pseudo-Hutchinson’s sign describes individuals with black or brown discoloration of the proximal nail fold secondary to non-melanoma pigmented lesions or other etiologies [2]. We expand the observations of proximal nail fold discoloration by not only describing a female with non-melanoma-associated black discoloration of her proximal nail fold (pseudo-Hutchinson’s sign), but also by reporting two males with either green or red-purple subungual pigmentation that extends to involve the proximal nail fold of the affected nail plate. Informed consent and photographing consent was obtained from all patients.

## Case presentation

**Figure 1:**
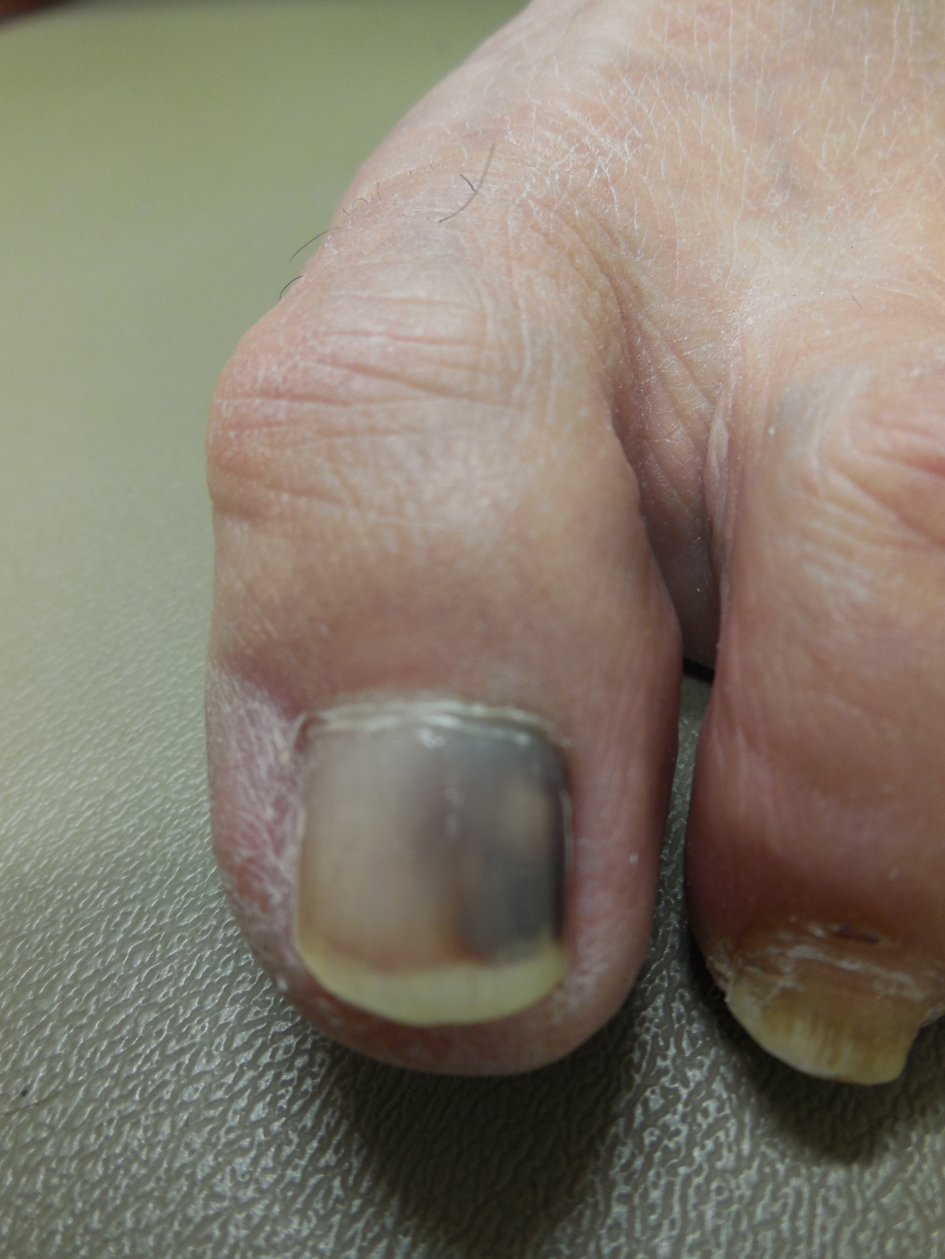
A 74-year-old female's left great toenail with black discoloration Superior view of a 74-year-old female's left great toenail with black subungual discoloration that involves the nail and extends to involve the proximal nail fold, described as pseudo-Hutchinson’s sign.

Case 1: A 74-year-old female presented for evaluation of asymmetric discoloration of her left great toe that had persisted for three months. She did not recall any prior history of trauma to the digit. A clinical examination showed black discoloration of the lateral half of her nail with similar pigmentation of the proximal nail fold (Figure 1).

**Figure 2:**
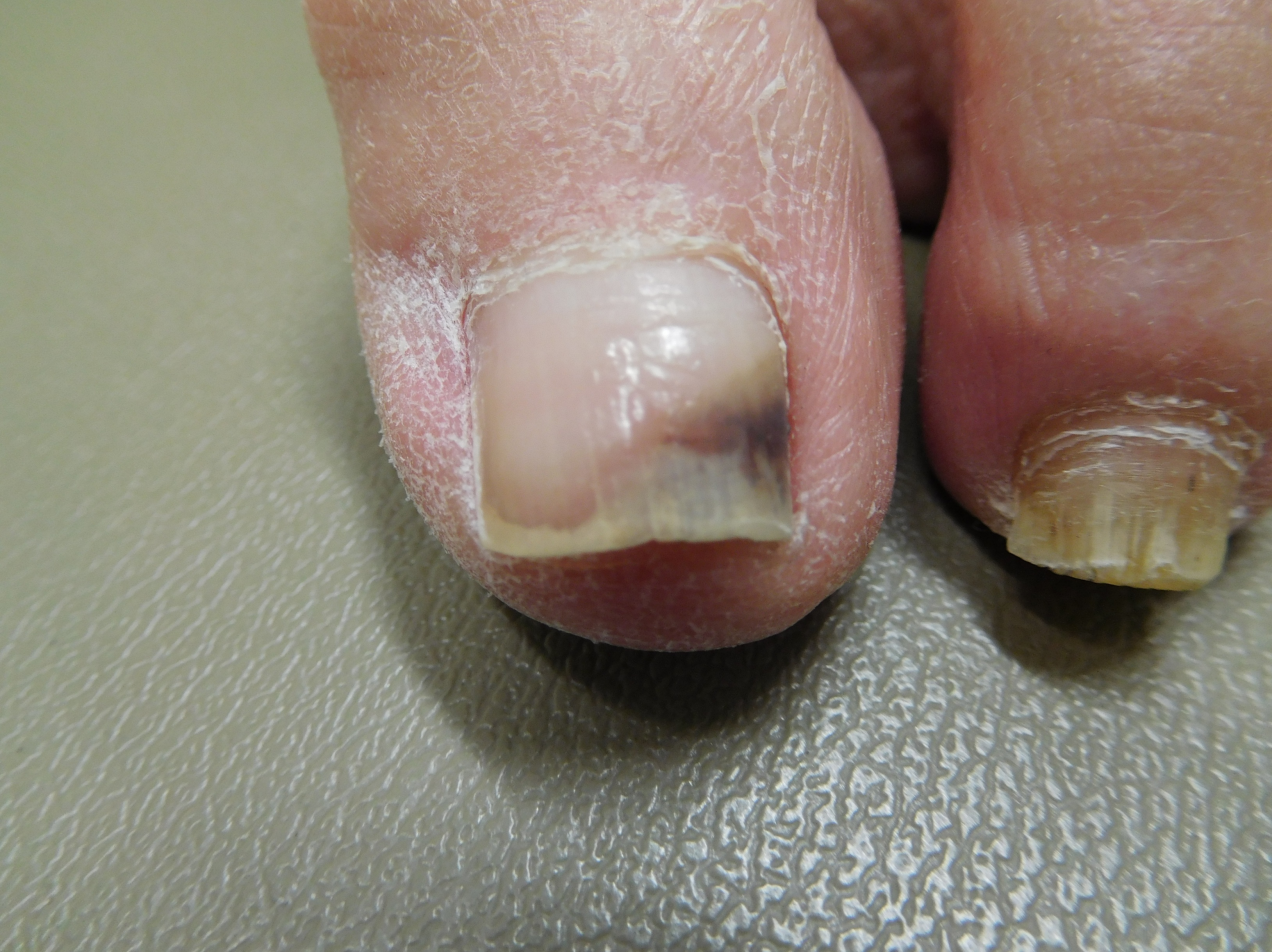
A 74-year-old female's left great toenail with improved black discoloration Superior view of her left great toenail six months later showing near complete resolution of the black subungual discoloration, which no longer involves the nail fold. The diagnosis is subungual hematoma.

Cutaneous microscopic examination of hematoxylin and eosin stained sections of nail clippings demonstrated hemorrhage into the nail plate. A periodic acid-Schiff (PAS) stain was negative for hyphae. Correlation of clinical presentation and pathology established a diagnosis of pseudo-Hutchinson’s sign. Follow-up examination six months later demonstrated absence of the pigment in the nail fold and 6 mm of normal proximal nail (Figure 2).

**Figure 3:**
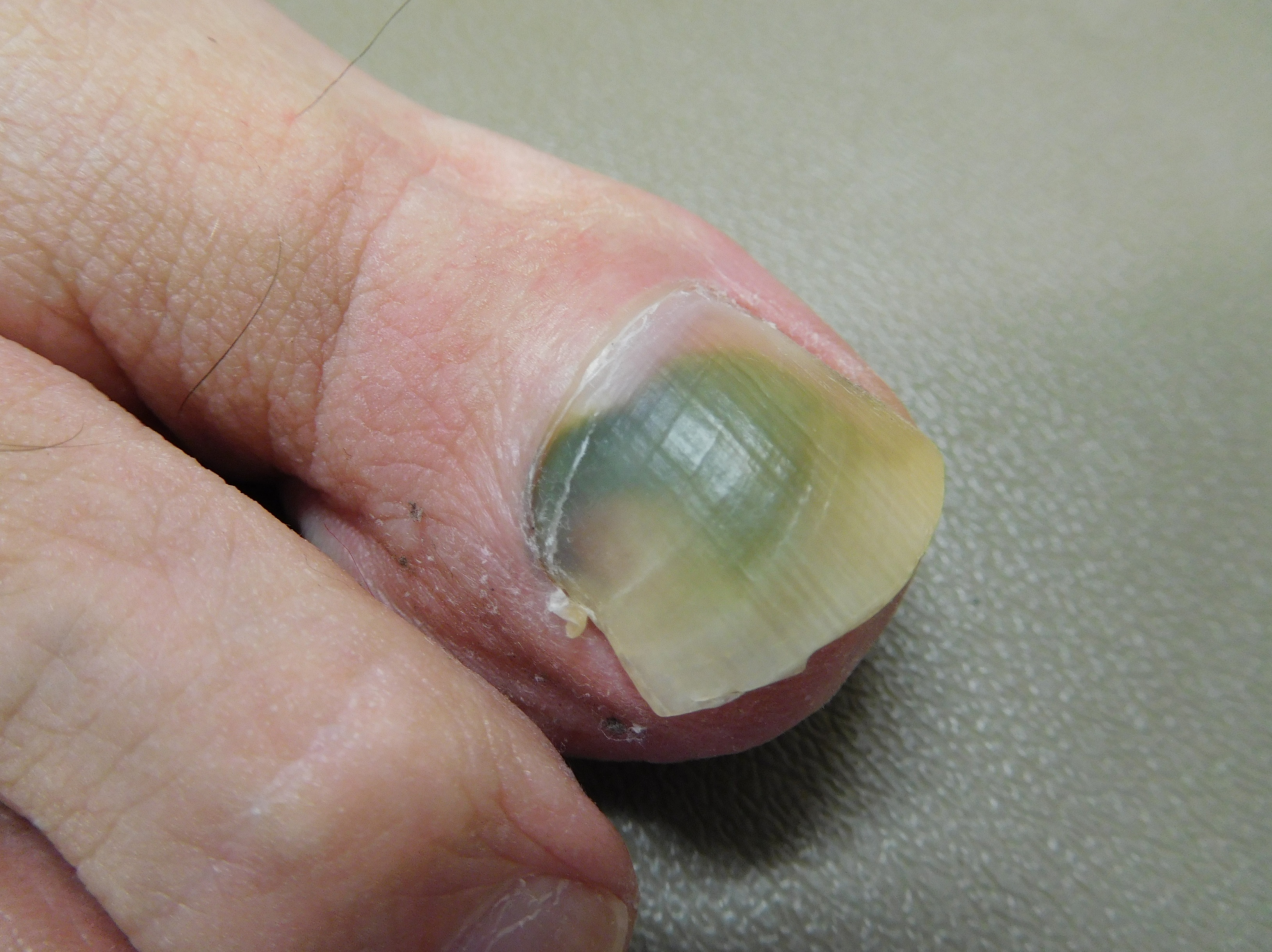
A 75-year-old male's right great toenail with green discoloration Superior view of a 75-year-old male's right great toenail with green subungual discoloration involving the nail and proximal nail fold.

**Figure 4:**
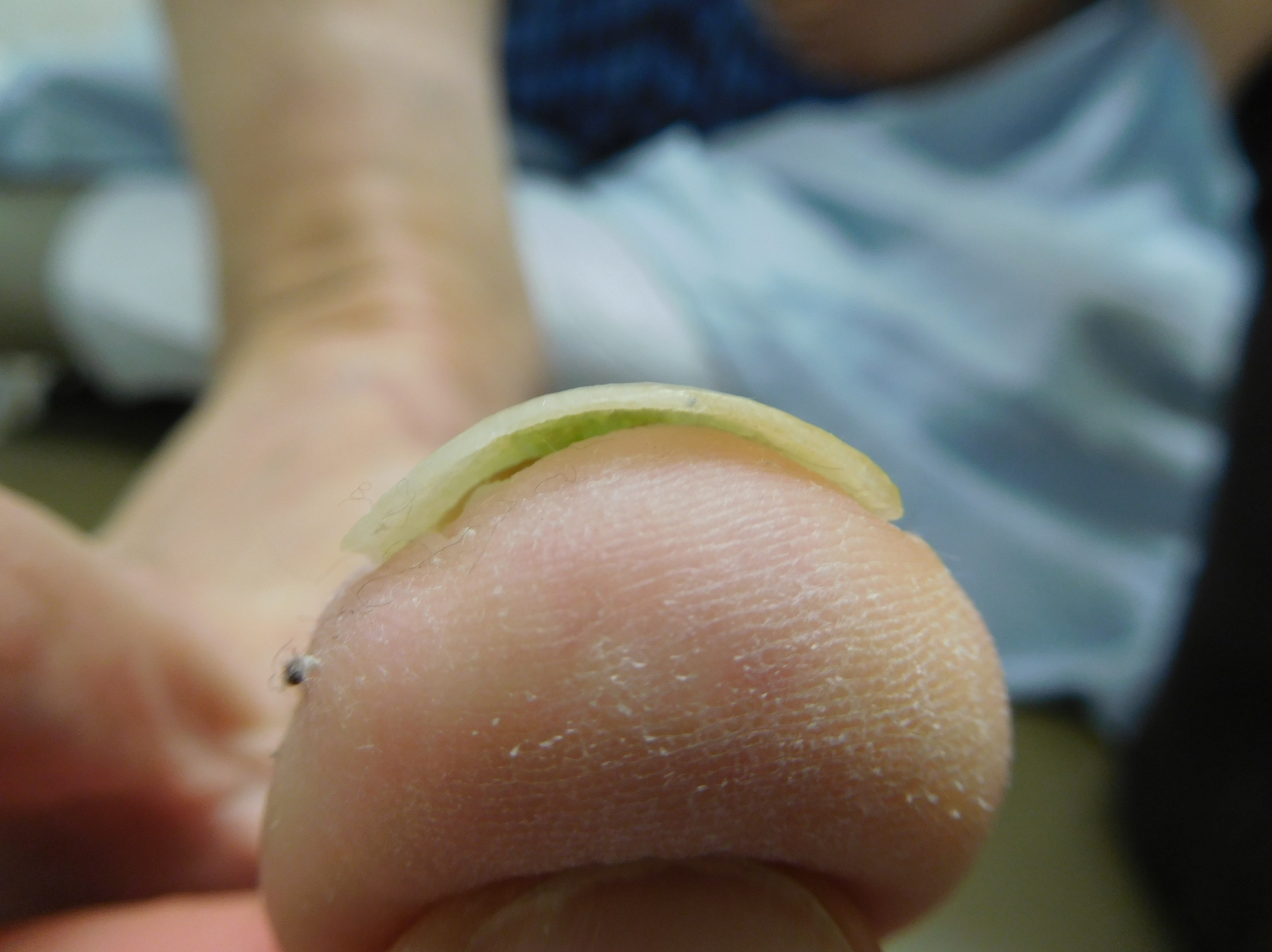
A 75-year-old male's right great toenail with green discoloration Frontal view of the right great toenail; the distal onycholysis permits visualization of green pigmentation on the underside of the nail plate.

Case 2: A 75-year-old male presented with asymptomatic discoloration of his right great toenail that persisted for six months. There was no history of trauma to the digit. A clinical examination showed green subungual discoloration with extension of pigment into the proximal nail fold (Figure 3). There was also distal onycholysis of the discolored nail (Figure 4). 

**Figure 5:**
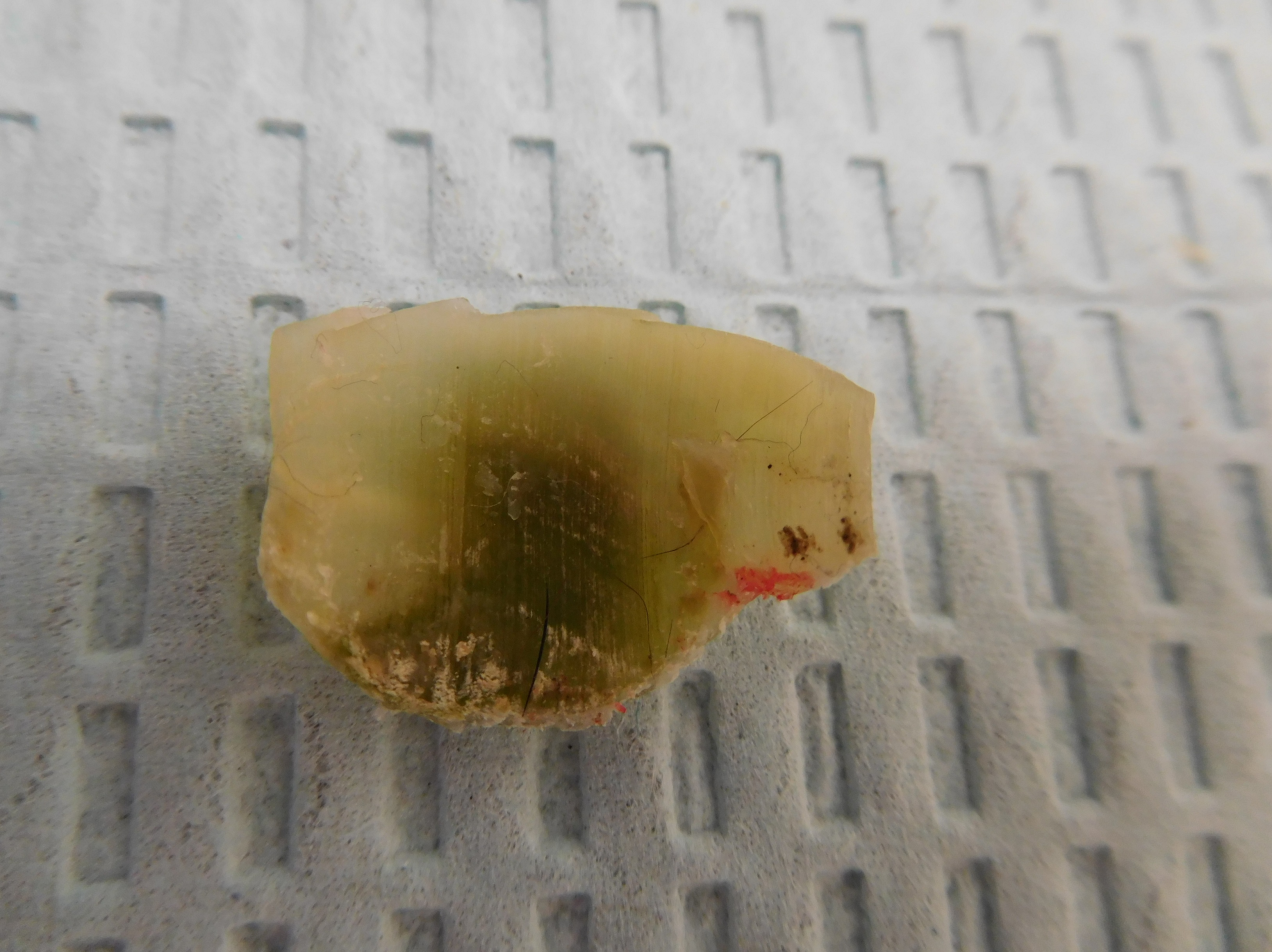
The right great toenail with green discoloration removed from a 75-year-old male The underside of the removed right great toenail demonstrates green discoloration. The diagnosis is green nail syndrome secondary to *Pseudomonas aeruginosa* infection.

Partial nail plate avulsion was performed with removal of the pigmented nail plate (Figure 5). Bacterial culture of the nail plate grew *Pseudomonas aeruginosa* (*P. aeruginosa)*. A microscopic examination of the nail plate showed focal subungual bacteria after a gram stain was performed; periodic acid-Schiff (PAS) stained sections were negative for hyphae.

Correlation of the clinical presentation, bacterial culture, and pathology established a diagnosis of green nail syndrome secondary to *Pseudomonas aeruginosa* (*P. aeruginosa)*. The exposed nail bed was treated with Floxin Otic 0.3% solution twice daily. A follow-up examination six months later showed no recurrence of green discoloration.

**Figure 6:**
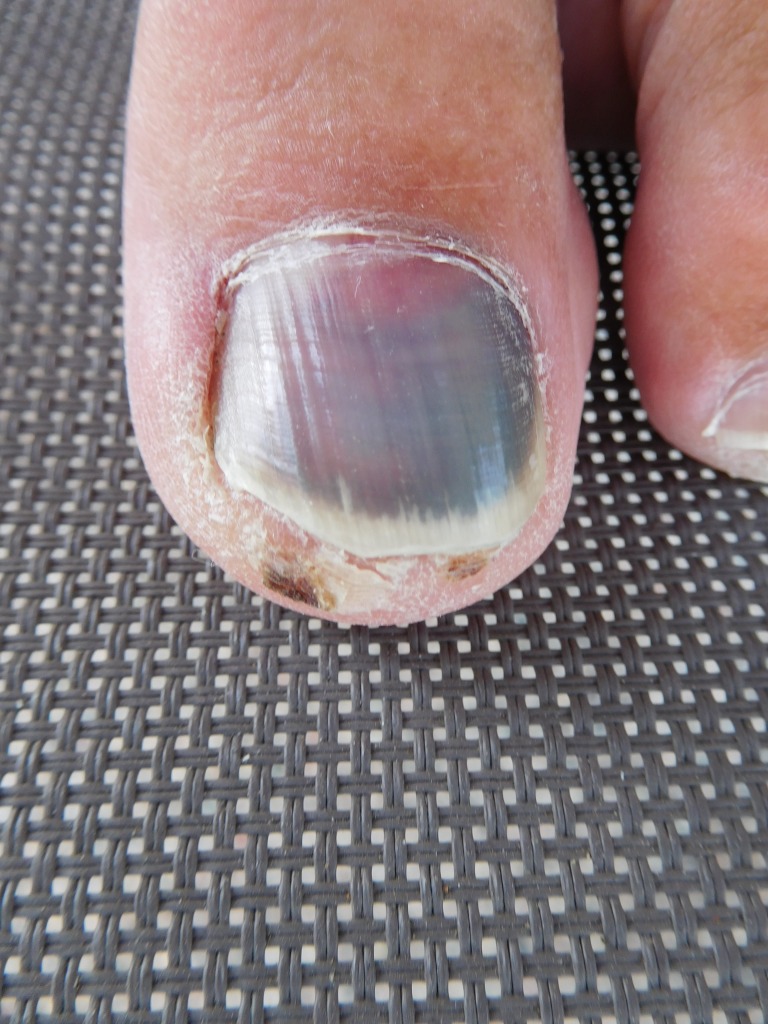
A 57-year-old male's left great toenail with red-purple discoloration Superior view of the left great toenail of a 57-year-old male demonstrating red-purple discoloration involving the nail and proximal nail fold.

**Figure 7:**
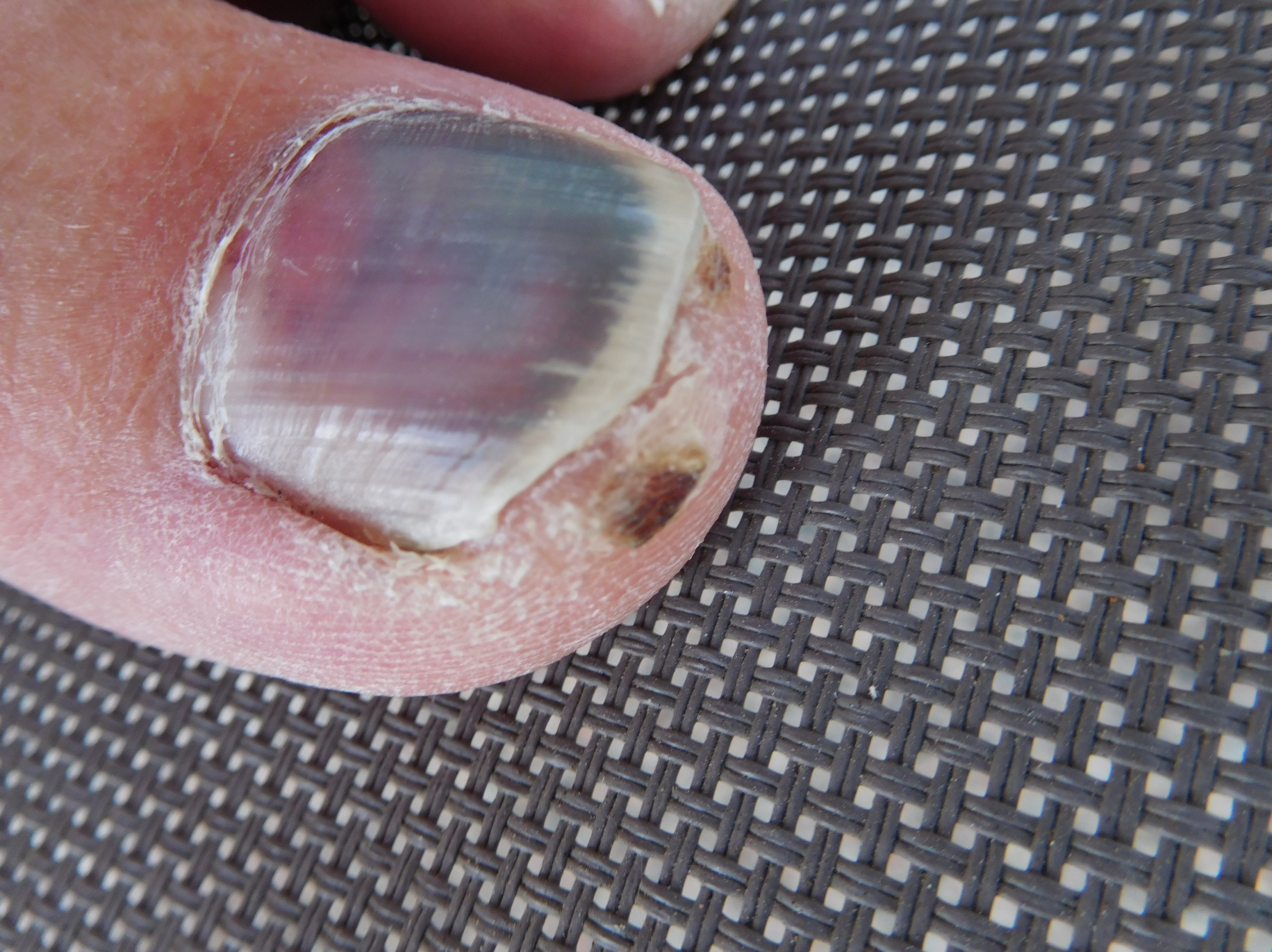
A 57-year-old male's left great toenail with red-purple discoloration Side view of the same left great toenail; talon noir (subcorneal hematoma) of the distal toe is also present. The diagnosis is acute subungual hematoma.

Case 3: A 57-year-old male presented for evaluation of discoloration of his left great toenail. Four weeks earlier he had participated in a 13.1 mile half-marathon. The subungual area thereafter became painful and discolored. A cutaneous examination revealed purple and red subungual discoloration involving not only the nail, but also the proximal and lateral nail folds. In addition, there was a subcorneal hemorrhage of the distal toe (talon noir) (Figures 6-7).

The distal overlying nail plate had become detached and partial nail plate avulsion was performed. Serosanguinous exudate drained during the procedure.

Correlation of the clinical presentation and response to treatment established the diagnosis of trauma-associated acute subungual hematoma. A follow-up six months later demonstrated normal proximal nail growth without pigmentation.

## Discussion

The nail plate is surrounded by the lateral and proximal nail folds. The eponychium (cuticle) is the distal portion of the proximal nail fold. It attaches to the surface of the nail plate [3].

Nail plate discoloration can occur secondary to multiple etiologies (Table 1) [2, 4-9]. Melanonychia is black-brown nail pigmentation caused by melanin deposition and develops due to either melanocytic activation or melanocytic hyperplasia. The causes of melanocytic activation include connective tissue disease (scleroderma, systemic lupus erythematosus) (SLE), drugs (antimalarials, azidothymidine (AZT), chemotherapeutics, phenytoin, steroids, sulphonamides), endocrine (Addison’s disease, acromegaly, Cushing syndrome, hyperthyroidism), infectious (AIDS, onychomycosis), inflammatory nail disorders (amyloidosis, chronic radiodermatitis, lichen planus, psoriasis), nutritional (vitamin B12 deficiency), physiological (pregnancy, racial), and trauma. Melanocytic hyperplasia causes include lentigo, melanocytic nevi, and melanoma. Melanonychia can also be associated with syndromes such as Laugier-Hunziker (pigmentation of oral mucosa, longitudinal melanonychia, and genital melanosis), Peutz-Jegher (pigmentation of lips and oral mucosa) and hamartomatous polyps [2, 4-5].

**Table 1:** Causes for diffuse nail plate discoloration The table presents a partial representative listing of etiologies for each nail plate discoloration.

Color	Etiology
Black/Brown	Melanocytic activation, melanocytic hyperplasia, subungual hematoma, and syndromes [2, 4-5]
Blue	AIDS, antimalarial agents, argyria, cyanosis, glomus tumors, hereditary arco labial telangiectasia, minocycline, Wilson’s disease [4-7]
Green	Fungal infection, *Pseudomonas aeruginosa* infection [4-5, 8-9]
Red	Carbon monoxide poisoning, chemotherapy, glomus tumor, subungual hematoma [4-5, 7]
White	Dermatological causes, drugs, infections, nutrition, poisoning, systemic diseases, and trauma [4-5]
Yellow	Drugs, onychomycosis, yellow nail syndrome [4-5]

Blue nails can be observed in patients with AIDS, argyria, cyanosis, glomus tumors, hereditary arco labial telangiectasia, minocycline use, and Wilson’s disease (an autosomal recessive disease secondary to a defect in the copper-transporting gene) [4-7]. *Pseudomonas aeruginosa* infection of the nail can cause green discoloration and is known as green nail syndrome [4-5, 8-9]. Conditions associated with red dyschromia include carbon monoxide poisoning, chemotherapy, glomus tumor, and subungual hematoma, as well as longitudinal erythronychia, red lunula and transverse red bands [4-5, 7].

Leukonychia causes include dermatological causes (alopecia areata, erythema multiforme, exfoliative dermatitis, lichen planopilaris, psoriasis, systemic lupus erythematosus), drugs (cyclosporine, cytotoxic agents, penicillamine, quinacrine, retinoids, steroids, sulphonamides), infections (leprosy, malaria), nutrition (malnutrition, pellagra, protein deficiency, zinc deficiency), poisoning (arsenic, fluoride), systemic diseases (cardiac failure, hypocalcemia, graft rejection, liver cirrhosis, liver failure, pneumonia, Raynaud’s disease, and trauma [4-5]. Yellow chromonychia can be caused by onychomycosis or yellow nail syndrome (a syndrome involving yellow dystrophic nails), lymphedema, and pleural effusions, or can be induced by antimalarial, gold-containing, hydroxyurea, penicillamine, and, tetracycline drugs [4-5].

To the best of our knowledge, proximal nail fold discoloration associated with subungual pigmentation has only been observed with black/brown, green and red/purple nails. Black or brown subungual pigment beneath the nail plate, when it extends to involve the proximal nail fold is referred to as Hutchinson’s sign when the etiology is associated with melanoma of the nail apparatus [2].

When the etiology of a black or brown discoloration of the proximal nail fold is secondary to pigmented non-melanoma lesions, it is referred to as pseudo-Hutchinson’s sign [1-2]. It can be part of a syndrome (such as Laugier-Hunziker syndrome or Peutz-Jegher syndrome) or an isolated finding [1]. In addition, non-melanoma lesions or conditions resulting in black pigmentation of the proximal nail and nail fold include congenital nevus, ethnic pigmentation, malnutrition, minocycline use, radiation therapy, tinea unguium infection, and trauma.

Green nail syndrome describes a green discoloration of the nails, also known as chloronychia. A culture of the affected nail plate will usually grow the bacteria *P. aeruginosa*. An antibiotic pigment produced by *P. aeruginosa, *pyocyanin, causes the green discoloration [9].

Often in green nail syndrome, there is distal onycholysis where the nail is separating from the nail bed. The bacterial pathogen may be colonizing the nail plate and localized. Systemic infection is usually not present.

Treatment of green nail syndrome frequently involves removal of the non-attached nail plate; this also results in the removal of the green pigment. Oral or topical antibiotics for which *P. aeruginosa* is susceptible may be used in treatment. Application of agents that permit drying of the nail bed are also effective in preventing recolonization, as *P. aeruginosa* is unable to colonize dry environments [8].

Subungual hematomas are typically associated with trauma to the nail or distal digit. However, in some circumstances the patient may not recollect injuring the affected area. Acute subungual hematoma presents as a tender area with red discoloration. Chronic subungual hematoma presents with asymptomatic dark purple and black discoloration.

Symptomatic treatment of acute subungual hematoma may involve evacuation of the hematoma to provide pain relief. Older or chronic subungual hematomas no longer have free flowing blood and a partial or complete nail plate avulsion may be required to evacuate the hematoma [10]. Nail plate with or without nail bed specimen may be sent for pathologic evaluation in order to establish a diagnosis of subungual hematoma and exclude other possibilities.

Although green nail syndrome and subungual hematomas often only affect the nail plate, occasionally the pigmentation can extend proximally to involve the nail fold, as in our patients. Currently, a descriptive term for pigmentation of the proximal nail fold that is not black in color remains to be designated. Although a new nomenclature might be created to define this clinical presentation, it might be preferable to modify currently established terminology. Therefore, we suggest that subungual pigmentation that affects proximal nail fold and is not brown or black in color, be referred as pseudo pseudo-Hutchinson’s sign.

## Conclusions

Hutchinson’s sign refers to the presence of black or brown subungual pigmentation, involving the proximal nail fold and associated with melanoma. Pseudo-Hutchinson’s sign describes similar black or brown subungual pigmentation involving the proximal nail fold, but is associated with non-melanoma lesions. Proximal nail fold subungual discoloration not associated with melanoma can also be seen in green and red-purple nails, like in our patients. Subungual pigmentation that is not black or brown and involves the proximal nail fold lacks a descriptive term. Instead of creating a new terminology for this clinical observation of nail fold discoloration associated with subungual pigmentation, we recommend that this observation be referred to as pseudo pseudo-Hutchinson’s sign.
